# An IoT-Based Healthcare Platform for Patients in ICU Beds During the COVID-19 Outbreak

**DOI:** 10.1109/ACCESS.2021.3058448

**Published:** 2021-02-10

**Authors:** Itamir de Morais Barroca Filho, Gibeon Aquino, Ramon Santos Malaquias, Gustavo Girão, Sávio Rennan Menêzes Melo

**Affiliations:** 1 Digital Metropolis Institute, Federal University of Rio Grande do Norte 28123 Natal 59078970 Brazil; 2 Department of Informatics and Applied MathematicsFederal University of Rio Grande do Norte 28123 Natal 59078970 Brazil

**Keywords:** Healthcare, Internet of Things, COVID-19, remote monitoring, platform

## Abstract

There is a global concern with the escalating number of patients at hospitals caused mainly by population aging, chronic diseases, and recently by the COVID-19 outbreak. To smooth this challenge, IoT emerges as an encouraging paradigm because it provides the scalability required for this purpose, supporting continuous and reliable health monitoring on a global scale. Based on this context, an IoT-based healthcare platform to provide remote monitoring for patients in a critical situation was proposed in the authors’ previous works. Therefore, this paper aims to extend the platform by integrating wearable and unobtrusive sensors to monitor patients with coronavirus disease. Furthermore, we report a real deployment of our approach in an intensive care unit for COVID-19 patients in Brazil.

## I. Introduction

There is a growing trend in the medical field to minimize the need for hospitalization, moving several health care procedures from hospitals (hospital-centric) to patient’s homes (home-centric) [1], [2]. This strategy has been praised mainly due to its potential for improving patient’s wellness and treatment effectiveness for a wide range of health conditions [3]–[5]. It can also reduce the costs of the public health system worldwide and its efficiency, which in the last decade has been challenged by the population aging and the rise of chronic diseases [6], [7]. Furthermore, the current COVID-19 outbreak has exposed the importance of rapidly scaling the health system and keeping at home patients who are high-risk but not severe enough to stay hospitalized [8].

Internet of Things (IoT) provides the scalability required for this purpose, supporting continuous and reliable health monitoring on a global scale. This paradigm is increasingly becoming a vital technology in healthcare [9]. Furthermore, the recent progress in low-power consumption, miniaturization, and biosensors has revolutionized the process of monitoring and diagnosing health conditions, bringing comfort, personalization, and effectiveness through unobtrusive healthcare devices [10], [11].

This paper extends the authors’ previous works [12], [13] by instantiating the Reference Architecture for IoT-based Healthcare Applications (RAH) for healthcare applications for the context of the COVID-19 outbreak. We show how wearable and unobtrusive sensors can be integrated into the proposed platform and used to collect and process patient data to promote rapid clinical interventions while preventing contagion between clinical staff and infected patients. Finally, we report the results from a real experience, which used our approach to develop and deploy a system used by the intensive care unit (ICU) for COVID-19 patients in Brazil.

Therefore, this paper aims to extend the platform proposed in [12], initially designed for patients’ de-hospitalization, by including wearable and unobtrusive sensors to monitor patients with coronavirus disease. We developed software components guided by the Reference Architecture for IoT-based Healthcare Applications [14] for interoperability with existing multiparametric monitors in a real intensive care unit (ICU) for COVID-19 patients in Brazil. By describing the engineering process and the application deployment steps performed in this experience, we provided relevant guidelines to practitioners and researchers concerned with IoT-based Healthcare Applications. Finally, to the best of our knowledge, we could not find any other work that reports similar experiences on the extension, development, and deployment of IoT applications in a real ICU.

In order to provide a better understanding of this paper, we included in table 1 a list of abbreviations used and their meaning.

**TABLE 1:** Abbreviations Used in This Manuscript

Abbreviation	Meaning
6LoWPAN	IPv6 over Low -Power Wireless Personal Area Networks
API	Application Programming Interface
CoAP	Constrained Application Protocol
COVID-19	Corona Virus Disease
CSV	Comma-separated values
ECG	Electrocardiogram
EDF	Earliest Deadline First
EHR	Electronic health record
GPRS	General Packet Radio Service
HL7	Health Level 7
HTTP	Hypertext Transfer Protocol
ICU	Intensive Care Unit
IDC	IoTDataCollector
IEC	International Electrotechnical Commission
IEEE	Institute of Electrical and Electronics Engineers
IoT	Internet of Things
IPv6	Internet Protocol version 6
IrDA	Infrared Data Association
JSON	JavaScript Object Notation
MQTT	Message Queuing Telemetry Transport
NBP	Non-invasive Blood Pressure
NFC	Near Field Communication
NoSQL	Not Only SQL
PHR	Personal Health Record
RAH	Reference Architecture for IoT-based Health care Applications
REST	Representational State Transfer
RFID	Radio-Frequency Identification
RSU	Remote Service Unit
SLR	Systematic Literature Reviews
SSF	Smallest Slack Time First
SWF	Smallest Workload First
VLAN	Virtual Local Area Network
WIFI	Wireless Fidelity
WSN	Wireless Sensor Network
XLSX	XML Spreadsheet
XML	Extensible Markup Language
XML-RPC	Remote Procedure Call

This paper is structured as follows: in Section II, we report a literature review presenting related works that address technologies, cases, and approaches related to the remote monitoring of COVID-19 patients. Section III describes the proposed IoT-based healthcare platform in the context of COVID-19, while the Section IV describes the implementation and deployment of a healthcare solution based on this architecture for a COVID-19 ICU. Finally, in Section V we present the concluding remarks.

## II. Related Works

Before proposing a new healthcare platform, we performed the review, presented by Barroca and Aquino [15], based on the Systematic literature reviews (SLR) method [16]. Its goal was to comprehend the current state and future trends in IoT-based healthcare applications. Thus, the research questions that addressed the review were related to the main characteristics (requirements), protocols, challenges, and opportunities related to these applications.

Regarding the main characteristics of these applications, we collected their functional and non-functional requirements from the studies. Therefore, the requirements described in the papers are the patient’s body and environment monitoring.

The sensors attached to the patient’s body (body monitoring) are the pulse oximeter, heart rate, galvanic skin, transpiration, muscle activity, body temperature, oxygen saturation, blood pressure, airflow, body movement, blood glucose, breathing rate, and ECG [11], [17]–[22]. The sensors deployed in the patient’s environment (environment monitoring) capture data from temperature, light, humidity, location, body position, motion data, SPO2, atmospheric pressure and CO2 [23]–[26].

The nonfunctional requirements described in the papers are scalability, reliability, ubiquity, portability, interoperability, robustness, performance, availability, privacy, integrity, authentication and security [17], [18], [23]–[26].

When it comes to protocols, the data collected from the studies showed two protocols categories: communication, concerning network protocols, and application, about data transfer protocols [26]–[32].

Thus, the communication protocols are 6LoWPAN, IEEE 802.15.4, Zigbee, Bluetooth, RFID, WIFI, Ethernet, GPRS, IEEE 802.15.6, 3G/4G, NFC, and IrDA. The application protocols are REST, YOAPY, HTTP, CoAP, XML-RPC, and Web Services. The studies presented that healthcare applications use HL7, XML, EHR, CSV, JSON, and PHR for the data format.

The studies showed that there are many challenges related to IoT-based healthcare applications, such as data storage and management (e.g., physical storage issues, availability, and maintenance), interoperability and availability of heterogeneous resources, security, and privacy (e.g., Permission control, data anonymity, etc.), unified and ubiquitous access area [33].

Moreover, the authors highlight the interoperability challenge since there have been different studies and proposals for patient monitoring at the hospital or home. It is still missing a shared goal to produce an interoperable system adopting open healthcare standards, such as HL7, and a seamless framework to be easily deployed in any given scenario [34]. Another challenge is how to provide constant monitoring, precise sensing, interoperability, and how to make the whole process unobtrusive for patients.

A few frameworks regarding healthcare IoT-based monitoring can be found in literature. These proposals can encapsulate a large variety of technologies such as cloud computing and fog computing. Following, we detail some of these works.

The work presented in [35] proposes a mobility aware framework that encloses concepts like Cloud, Fog and Edge Computing working together with an IoT layer called Mobi-IoST. The authors consider a scenario where a sensing layer composed of several sensors like body temperature, heart rate, blood pressure can be used to measure vital signs of a patient. From that, this information can be gathered and become available to medical staff even during the patient transfer in an ambulance. The main problem is how to provide this data while moving. Data connection can be frequently lost and the availability and consistency of the information can be compromised. The authors propose to solve this by means of a framework comprised of an IoT Layer, Fog Layer, Cloud Layer and Edge Layer.

In the scenario presented, Remote Service Units (RSU) serve as large cell based stationed in several points during the ambulance path will form the Fog layer. From that, it is possible to send the patient’s data that can be relayed to the hospital. On the other side, a mobility analysis module on the Cloud layer can reduce the arrival time of the ambulance by predicting less traffic on a certain path. The authors also present algorithms for path prediction that can run inside the RSU or inside the Cloud and a delay and power model is provided and analysed. Finally, the paper shows that the framework proposed presented an improvement of 10-18% compared to other solutions. Also, the Mobi-IoST reduces the delay and power consumption by 23-26% and 37-41% respectively when compared to existing systems.

The authors in [36] propose a fog-based framework in order to provide real-time task scheduling. The main issue is the fact that some information on healthcare is time sensitive such as ECG. By using fog cloud servers, the time constraints become even harder to fulfill. Therefore, a healthcare awareness cost-efficient task scheduling (HCCETS) framework is proposed. Task sequencing and Task schedule issues are considered for each critical task concerning different types of information such as heartbeat, ECG and others. In order to deal with distinct situations, the authors made use of more than one classic task scheduling algorithms such as Earliest Deadline First (EDF), Smallest Slack Time First (SSF) and Smallest Workload First (SWF). For each situation, all algorithms were applied in order to observe the best one suited. Experiments using a fog based cloud system were performed observing data storage and latency using public datasets. For each scenario, three configurations of Fog Server were used, each one with a different cost. The performance evaluation presented that the framework solution proposed outperforms the existing methods regarding cost.

In [37], the authors present a novel approach for adaptable time-critical cloud systems. This proposal is based on an application infrastructure co-programming model and features application composition by means of better programmability and controllability. Considering three time/critical applications, the papers show that the SWITCH architecture is capable of dealing with monitoring of services through adaptive tools, dynamic real-time planning and QoS applications. However, no specific healthcare application was used as benchmark.

Considering the global events related to the COVID-19 pandemic, a more thorough and updated healthcare platform review was performed. Here are presented work that shows interesting ideas on this issue even before the pandemic. Several papers also show technological alternatives regarding ICU patient care, homecare, transmission tracking, and symptom analysis on people without COVID-19 confirmation.

Moreover, it is important not to lose sight of the scientific developments presented before the pandemic. These studies have led to our current state-of-the-art. Nowadays, advanced technology has provided the industry with a new way of development. The usage of new concepts such as the Internet of things, big data, artificial intelligence, and cloud computing has made several industrial endeavors a new milestone known as the Fourth Industrial Revolution or simply Industry 4.0.

**FIGURE 1.**
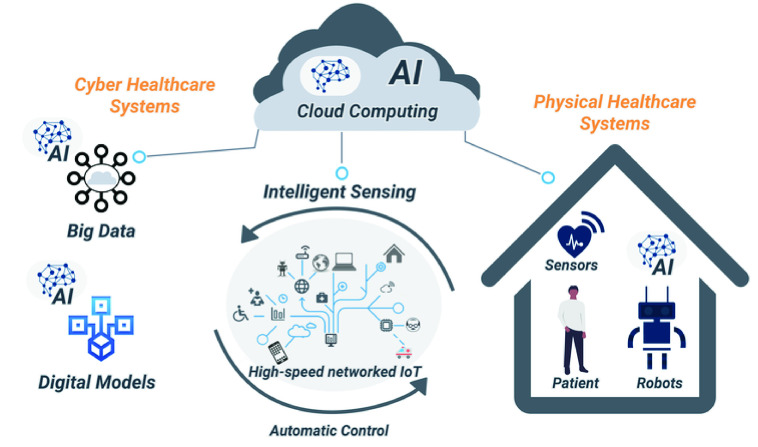
Technology integration in a Healthcare 4.0 scenario. Adapted from Yang et. al [38].

According to Yang *et al.* [38], in the context of Industry 4.0, the medical subarea has improved dramatically with new technological support coming from these emerging fields. This is called Healthcare 4.0. It is defined as an ever-flowing (and yet revolutionary) change in the whole healthcare scenario, including medical equipment, hospital care, home care, logistics, and a healthy living environment. This Healthcare 4.0 encapsulates the idea of a shift of design paradigm considering sensing data fusion and data interpretation. It relies heavily on constant monitoring of the human condition and communication between systems. This outline creates a scenario where Physical Homecare Systems meet Cyber Homecare Systems, as shown in Figure 1. The entire environment comprises distinct areas of expertise, such as big data, IoT, cloud computing, etc.

Regarding sensing, it is important to note that sensors must have properties such as bendability, stretchability, and ultrasensitivity. Besides, they must become unobtrusive and conformal to human skin to replace regular human monitoring equipment such as an ECG.

Regarding specific works that deal with healthcare scenarios (in or out of a hospital), a few proposals use IoT infrastructure and cloud computing to provide a robust solution. Next, these works are discussed.

Some works, such as the ones presented [39]–[41], describe an IoT-based framework for remote patient monitoring. These papers typically detail an IoT system architecture based on the three-layer modeling: a hardware module comprised of a few vital sign sensors, a gateway layer responsible for collect data, store it and make available for the higher level, which is the application layer. There are variations in the communication layer regarding the usage of different protocols, but overall, they follow similar architecture, which has become common in many new works like this.

**FIGURE 2.**
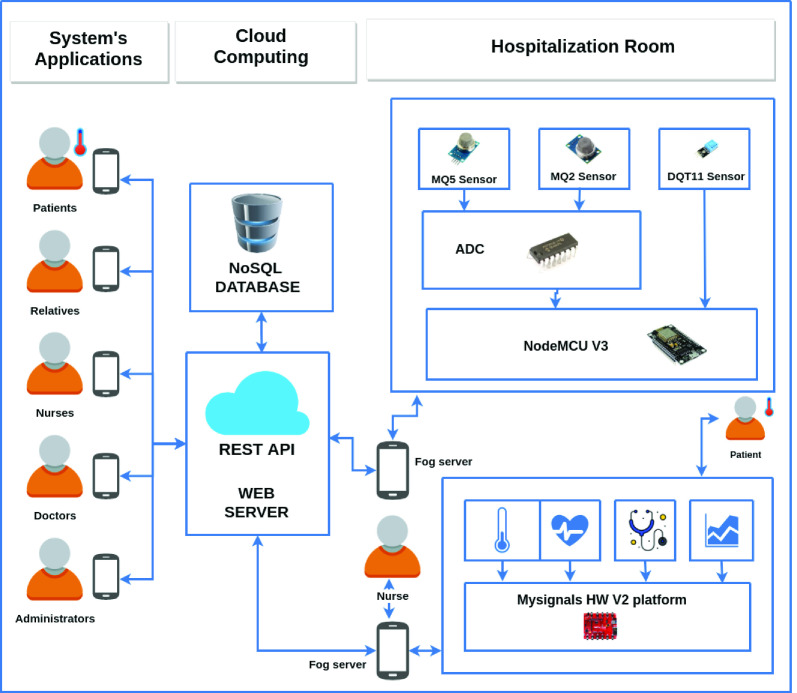
Home hospitalization system. Adapted from Hassen et. al [42].

Hassen, Ayati, and Hamdi [42] present a proposal for a home hospitalization system that uses IoT, fog, and cloud computing. The system architecture considers local monitoring and remote monitoring using sensing units and a mobile application. This mobile application acts as the Fog server and allows for the analysis of environmental aspects inside the patient’s room while the sensors capture the vital signs. All this information is provided for nurses and medical staff. A NoSQL database provides data persistence in order to deal with relational database problems regarding the heterogeneity of data, for instance. Figure 2 resumes the proposed architecture and comprises the actors in the system using the appropriate mobile application, the database, the web server, and the environmental and vital signs sensing units. It is important to note that this work presents a complete solution: from the sensing layer to the application layer. However, there is no discussion regarding interoperability with other systems already in use in a hospital environment. In this proposal, the authors assume a complete replacement of equipment. This hurts the argument of a low-cost system since it can have a higher price than a system that complements an already established monitoring system.

Ding *et al.* [43] present an extensive discussion about wearable sensing and telehealth technology as potential usage in the COVID-19 pandemic. The paper’s focus is divided into three main categories: i) the usage of wearable devices to monitor population risk; ii) patient monitoring through unobtrusive sensing and; iii) telehealth technologies for remote monitoring. It discourses extensively over several alternatives for vital sign wearable monitoring regarding oxygen saturation, respiratory rate, and lung sounds. These wearable solutions can be used in clothes, pulse (like watches), epidermal thermal sensors, or breathing belts. Smartwatches are also presented as wearable sensors to provide blood pressure monitoring. Simultaneously, small patches can be used specifically to monitor temperature and cough with the aid of smartphone applications (using its microphone). These solutions are presented as the state of the art resources to monitor COVID-19 patients. Outside a hospital environment, the authors also discuss the use of Contact Tracing technology to obtain reliable virus transmission results. This is also important to remote physiological monitoring outside the hospitals for patients in quarantine at their homes. Overall, this paper condensates several wearable unobtrusive monitoring solutions and provides a technology road map for multi-parameter physiological monitoring by means of wearable and unobtrusive sensors for COVID-19 of other possible pandemics.

Currently, humanity’s greatest efforts are focused on COVID-19 related solutions. Regarding healthcare solutions, there are several proposals of methods and mechanisms related to transmission tracking and monitoring of diagnosed and not diagnosed people living in areas where the COVID-19 cases have not yet dropped. There are already a few papers showing how to use IoT-based platforms to help such COVID-19 related scenarios. These proposals may vary from wearable devices to monitor possible symptoms. For instance, a headset-like device has been detailed by Stojanović [44] that can be used alongside a mask. This device can monitor Respiration Rate and alert users and medical staff about dangerous levels. Other works [45], [46] focus on monitoring through smartwatches to provide information about location and symptoms to help not only diagnose but also to calculate the degree of contamination.

In the end, these papers show that the efforts of combining IoT-based platforms, cloud computing, and mobile application development can be largely used to provide insights and to help COVID-19 diagnosis.

**FIGURE 3.**
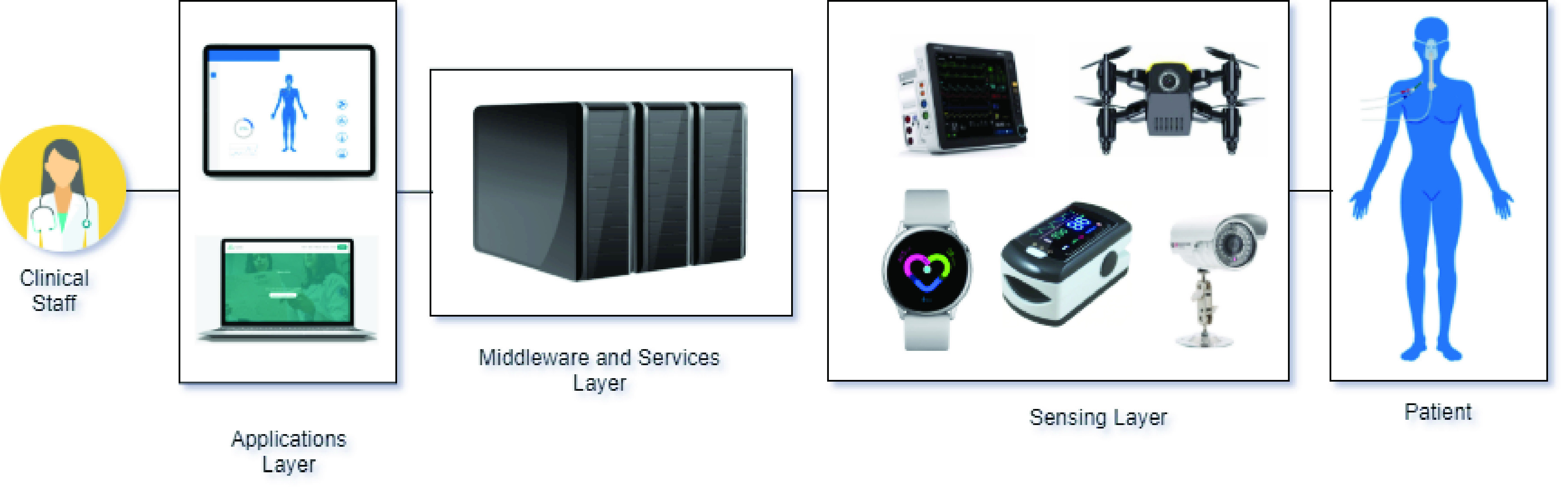
Examples of sensors and applications.

The examples presented in Figure 3 are IoT-based healthcare applications, and sensors can help monitor patients with COVID-19 and prevent contagion: wearables and applications for receiving and processing data of patients, promoting rapid clinical interventions; drones and cameras to perform temperature monitoring people of crowded areas; multi-parametric monitors with sensors to ECG, respiration rate, temperature, oxygen saturation, and applications to watch the patient’s health status; and proximity sensors and mobile applications to ensure a safe distance between individuals.

Finally, considering the need for intelligent monitoring of patients, we developed PAR, an IoT-based healthcare platform present in Section III. It is important to note that, to the best of our knowledge, none of the papers presented in this section detail the deployment of the actual healthcare monitoring system in a hospital. This is one of the main contributions of our paper.

## III. Proposing an IoT-Based Healthcare Platform

This section presents the IoT-based healthcare platform, named PAR, describing the methodology that guided its development, the design and requirements aspects, the actors, and use cases. It also explains the platform’s architecture considering its modules, their relationship, components and protocols.

### A. Methodology

**FIGURE 4.**
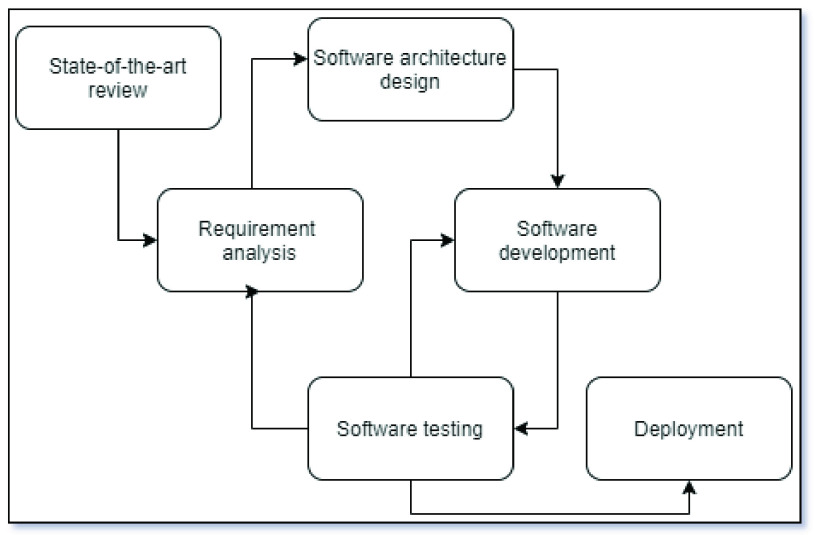
The methodology used to develop PAR.

The methodology used to develop PAR, presented in Figure 4, consisted of the following steps: State-of-the-art review, Requirement analysis, Software architecture design, Software development, Software testing, and Deployment.

Eight people participated in this development: the software architect of PAR, responsible for conducting and documenting the instantiation process; five developers responsible for supporting the requirements elicitation and implementation of PAR; and two registered nurses assisting the domain analysis activity.

In the step of the State-of-the-art Review, we performed and updated a review aiming to comprehend the current state and future trends for IoT-based healthcare applications and find areas for further investigations. With this study, it was possible to determine the main characteristics, functional requirements, quality attributes, and nonfunctional requirements, challenges, and opportunities of these applications. The findings of this review are presented in Section II.

In the Requirement analysis step, we created the requirements document containing functional and nonfunctional requirements of PAR. Besides, we identified in the Reference Architecture for IoT-based Health care Applications (RAH) [14] what strategies we would use to satisfy the requirements of PAR [13]. We used these outputs in the next step, Software architecture design, focused on the design and documentation of PAR’s software architecture.

We codified the software in the Software development step using mainly Java and based on the designed software architecture. In the Software testing step, we performed software testing using functional, regression, and stress tests.

Finally, in the Deployment step, we deployed the developed solution in the Intensive Care Unit (ICU). The following sections present these requirements, design, and deployment in a real context.

### B. Design Issues and Requirements

The proposed IoT-based healthcare platform’s primary goal is to provide remote monitoring for patients in a critical situation. This platform is IoT based and integrates patients, physicians, and ambulance services to promote better care and fast preventive and reactive urgent actions. It addresses challenges like interoperability, security, performance, and availability. Regarding requirements, this platform has *Remote Patient and Environment Monitoring, Patient Healthcare Data Management, Patient Health Condition Management, and Emergency and Crisis Management*.

The *Remote Patient and Environment Monitoring* involves the acquisition of data from sensors attached to the patient’s body and in the environment (patient’s home or ICU). The data acquired from the sensors are used by clinical staff (physician and nurses) for healthcare treatment and emergency alert purposes. Thus, the sensors attached to the patient’s body provide information about ECG, blood pressure and glucose, heart rate, oxygen saturation, temperature, breathing rate, and capnography. The environment sensors provide information about environment temperature, location with latitude and longitude, and humidity. This is important because controlling the environment’s temperature and humidity can directly affect the patient’s treatment. Regarding the location, it assists in the rapid response of the ambulance service. Therefore, since the patient in critical condition is at home and not in a hospital, which is a more controlled environment, this ambient information is of greater importance for effective healthcare and enriches the remote monitoring provided by this platform.

The *Patient Healthcare Data Management* records the patient’s data: name, gender, date of birth, contacts, address, family information, physician information (name and contacts), health insurance information, health situation, and the history of monitoring sensors and emergency alerts. These data are essential for physicians and nurses to understand patients’ current situation and history and facilitate the accurate monitoring of health treatment.

The *Patient Health Condition Management* considers the patient’s healthcare data, especially the health situation and history of the sensors’ monitoring data, to allow the definition of critical level values for the sensors, which are important to enable the rapid response in case of an emergency. It also defines rules to actions considering the settled critical levels for a patient and the related alerts.

Finally, the *Emergency and Crisis Management* address information about the patient’s health condition and the services that should be alerted in case of an emergency with a monitored patient in a critical situation. Since this patient is at home and not in a hospital, the efficiency of a rapid response in an emergency case can be the deciding factor between life and death.

**FIGURE 5.**
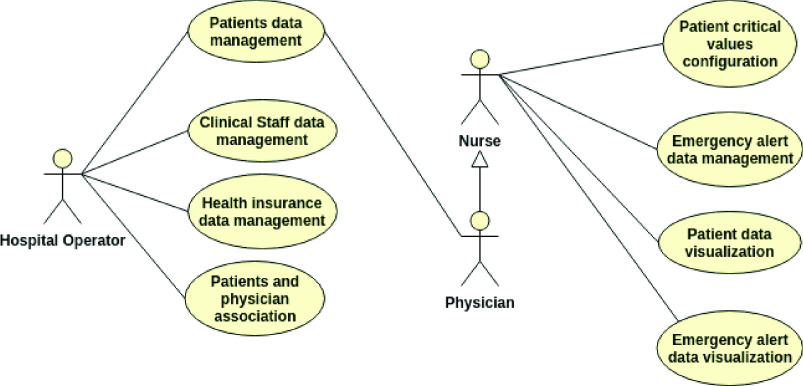
Healthcare platform use case diagram.

This platform comprises eight use cases that achieve the introduced requirements, presented by the use case diagram in Figure 5, and three actors: the hospital operator, physician, and nurses.

Considering the hospital operator actor, it interacts with the use cases related to Patient, health insurance, and clinical staff data, which are:

• Patient’s data management: it allows registering data related to Patient Healthcare Data Management mentioned earlier;
• Clinical staff data management: it allows to register data related to the clinical staff (physician and nurse). These data include: name, contacts, and specialty from physician and nurses;
• Health insurance data management: it allows to register data from health insurance;
• Patient and physician association: it allows to register the responsibility of a physician with a patient.

Regarding the physician and nurse actors, they can use the patient’s data management and interact with other use cases related to Remote Patient and Environment Monitoring, Patient Healthcare Data, Patient Health Condition Management and Emergency and Crisis Management, such as:

• Patient’s critical values configuration: it allows the definition of critical level values for sensors attached to the patient’s body, which are considered in alerts and notifications. Critical values can be configured in a personalized way for each patient or for a group of patients;
• Health data from patient: it allows the visualization of real-time health data from the sensors deployed in any patient’s body and environment;
• Patient data visualization: it allows the visualization of real-time health data from the sensors deployed in a single patient’s body and environment;
• Emergency alert data management: it allows the notification and alerts to be presented and managed by physicians and nurses;
• Emergency alert data visualization: it allows real-time viewing of emergency alerts for each monitored patient and is related to Emergency and Crisis Management.

Thus, the proposed platform was developed considering the presented requirements, actors, and use cases. It provides integration between patients and clinical staff for the efficient healthcare of patients in critical condition.

**FIGURE 6.**
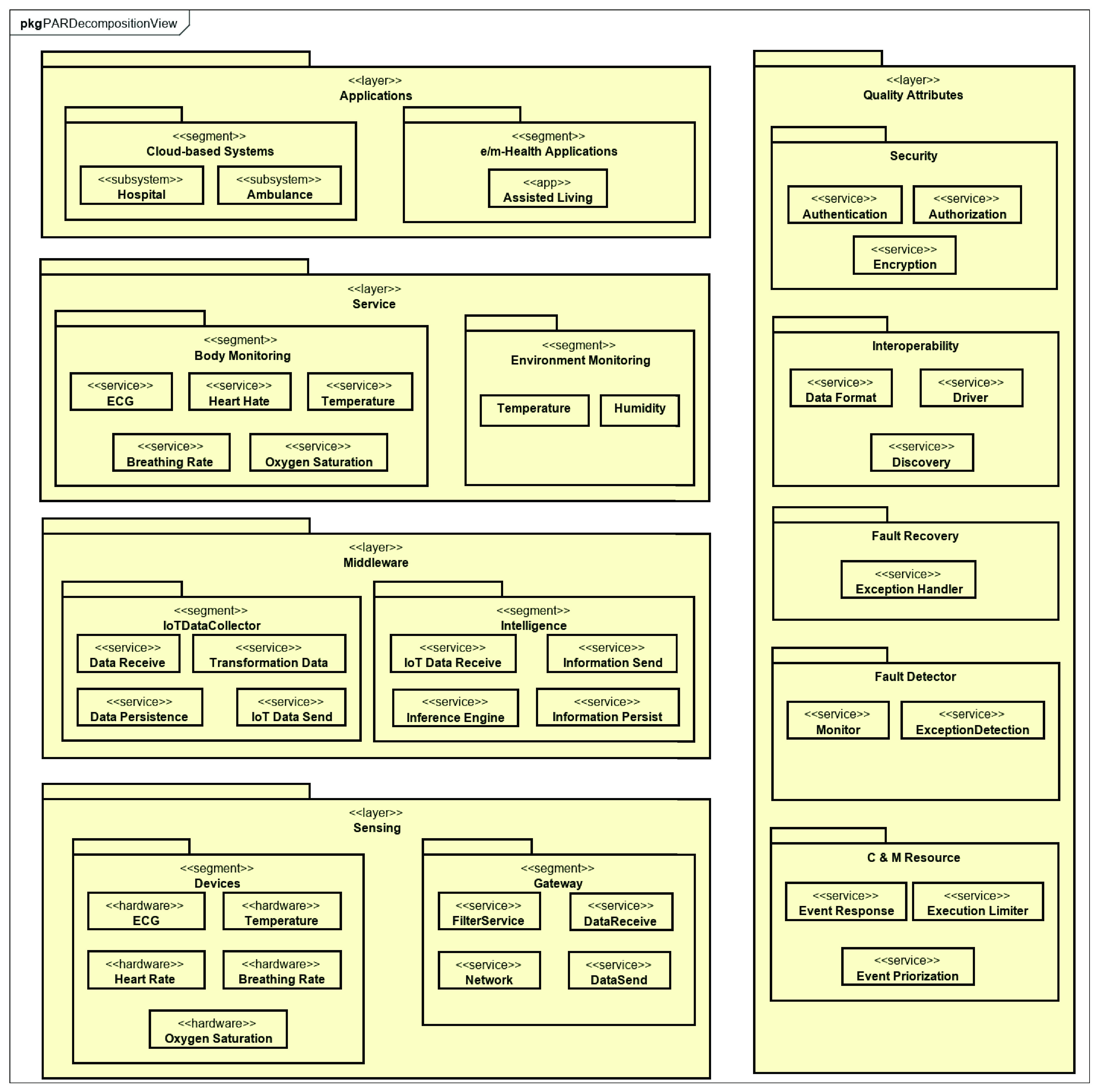
PAR decomposition view as an instance of RAH.

**FIGURE 7.**
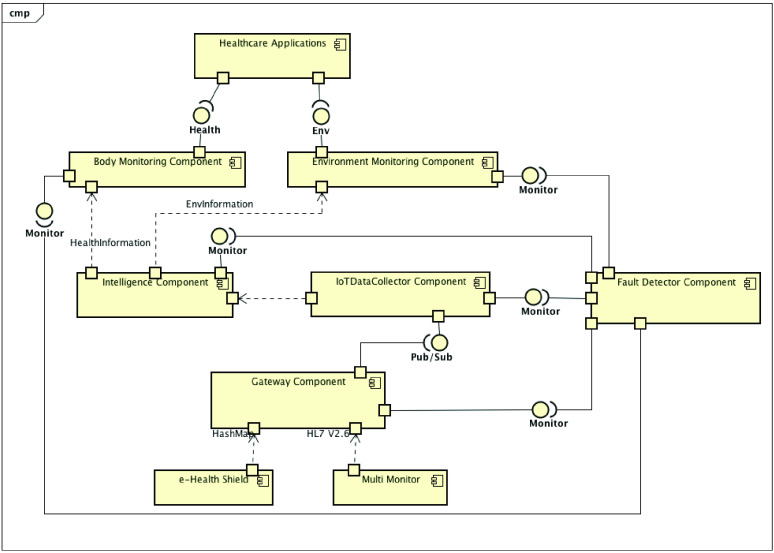
PAR component and connector view.

Figure 6 shows the PAR decomposition view presenting this platform in a fragmented way and detailing its services, and Figure 7 offers the PAR component and connector view. We created the software architecture of PAR, considering the requirements and instantiating services from the Reference Architecture for IoT-based Healthcare Applications (RAH) proposed by Barroca and Aquino [13], [47].

The data flow presented in this view starts with the devices sending the raw data (HL7 V2.6 and HashMap) to the Gateway. The Gateway packages the data; it defines the packet headers and sends them to the IoTDataCollector (IDC) that receive the data packets, persist, and treat them.

Hence, the Intelligent Component will apply its rules of inference about the IoT Data, so that this data is semantically understood and presents information about the health status of a patient. The service layer components (Body and Environment Monitoring) act as interfaces that abstract the requests for information about patients’ health and the environment in which they are accommodated. Finally, this information reaches the applications and is presented to the end-users of PAR.

### C. Modules Overview

**FIGURE 8.**
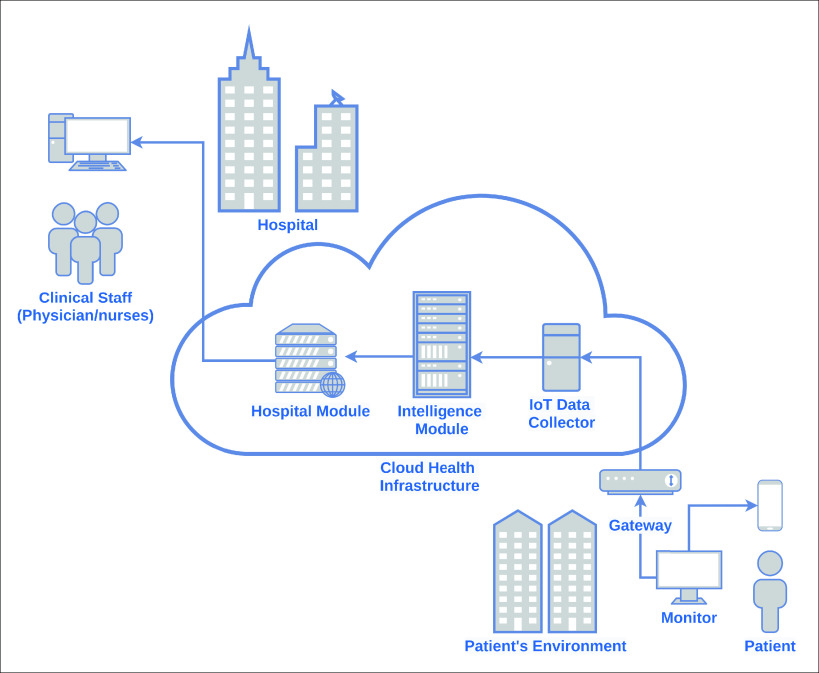
IoT-based platform to integrate patients, physicians and clinical staff.

The IoT-based healthcare platform, presented in Figure 8, comprises three parts: Patient’s Environment, Cloud Health Infrastructure, and Hospital. These parts address the solution’s functional requirements and work together to achieve remote monitoring and efficient healthcare for patients in critical condition.

The Patient’s Environment part is mainly composed of sensors that provide body and environment remote monitoring. The sensors attached to the patient’s body, described in the previous section, are part of a multi-parameter portable patient monitoring, which continuously measures the patient vital signs. The clinical staff configures this monitoring at the patient’s home and does not require patient intervention.

Regarding security and essential performance issues, the monitor agrees with the IEC standard, 60601-1-11:2015 (IEC 60601, 2015), which defines the basic safety and critical performance of medical electrical equipment medical electrical systems for use in the home healthcare environment. The environment monitoring sensors, described in the previous section, can be implanted in an ICU bed or the patient’s home.

Thus, this multi-parameter monitor is connected through the Internet to the Cloud Health Infrastructure module, as presented in Figure 4. It uses HL7 [48] as a standard for data streaming. The environment sensors are connected to a gateway through the 6LoWPAN protocol. Regarding 6LoWPAN, it is a protocol for Wireless Sensor Networks (WSNs) defined to enable IPv6 packets to be carried on top of low power wireless networks, specifically exploiting IEEE 802.15.4 protocol [34]. The Internet also connects this gateway to the IoT Platform of the Cloud Health Infrastructure part. The reason to use a gateway for the sensors is that they do not have interfaces for direct connection to the Internet.

Regarding the Cloud Health Infrastructure part, it is composed of the IoT Data Collector, the Intelligence Module, and the Hospital Module. These systems implement the use cases described previously. The features of each system are:

• IoT Data Collector: responsible for receiving sensors’ data. This is a challenge due to the huge number of devices integrated into this component, and its diversity, data formats, and protocols;
• Intelligence Module: configuration of the patient’s critical values for alerts displayed in the Hospital Information. These critical values are used in the rules defined by doctors and nurses on the platform itself. This system provides the possibility of defining the rules in a personalized way that is appropriate for each patient or, in general, for a group of patients, according to the medical team’s criteria. The rules are composed of a type of sensor, a value for the minimum safety limit, and a maximum safety limit value.
• Hospital Module: management of data from patients, clinical staff and health insurance, and the association between patients and physicians. It also provides mobile views for patient data and alerts visualization.

The data received by the IoT Data Collector are used by the Intelligence Module, which contains rules and creates derived information to be used by the Hospital Information System. The Intelligence Module uses Machine Learning and Analytics techniques based on the vast amount of data received to produce knowledge about the patient’s health behavior. This knowledge is then stored in this module, and users call upon the platform for specific advice as needed.

The platform can make inferences and arrive at a specific conclusion [49]. For example, critically ill patients, particularly those with hemodynamic instability signals, need a diagnosis, and immediate treatment. This condition presents itself with signs of tissue perfusion and impaired tissue oxygenation, which is usually detected by microcirculatory parameters or global hemodynamic measurements such as blood pressure and oxygen saturation in arterial blood [50].

When this Intelligence Module captures a critical value, it automatically generates an alert message to the Hospital Module and the clinical staff providing support for a specific decision on when and how to intervene. Thus, the patient’s state of the classification system is issued along with the monitoring values. Another example is if the sensors’ data show that the patient’s heart rate is zero, it can translate this as a heart attack. Therefore, this Intelligence Module also notifies the Hospital Module and the clinical staff.

Moreover, the Intelligence Module also provides an API to make the patient’s information available to authorized third party systems, using OAuth V2.0 [51], taking into account privacy and ethics. This API is composed of RESTFul Web Services [52] and uses JSON [53]. This API aims to make it easier to develop new solutions with the use of this data to promote innovation in the healthcare area. As a result, companies and researchers can benefit from this use.

The Hospital module is used by physicians, nurses, and clinical staff, and it uses the Hospital Information System. This system contains the patient’s records, including information about age, gender, name, contacts, family contact. It also provides real-time remote monitoring of patients in critical condition. Integrated with this Hospital Information System, there is a mobile app, which, in case of any problem with a patient, notifies the physician responsible. With this notification, the mobile app also presents the real-time situation and data from the sensors, such as ECG, blood pressure, blood glucose, heart rate, oxygen saturation, temperature, and breathing rate.

Regarding the environment monitoring provided by this platform, all data from the sensors - temperature, location, and humidity - are presented at the Hospital Information System and its mobile apps. This monitoring is essential because the environment’s temperature and humidity control can directly affect the patient’s health treatment.

There are important requirements that need to be addressed: privacy, security, interoperability, scalability, reliability, robustness, ubiquity, portability, performance, and availability. This platform uses encryption to ensure privacy. There is also a particular need for security, which is mainly guaranteed by authentication [54]. The IoT platform from the Cloud Health Infrastructure part assures scalability, integrity, portability, and interoperability between connected monitoring devices. These modules address the need for ubiquity, considering the defined rules and the customization feature.

Finally, the proposed platform and its modules aim to achieve good performance, robustness, reliability, and availability regarding patient monitoring information. Moreover, with the use of HL7 and an IoT data collector, we propose to solve interoperability issues, and with permissions controls and OAuth V2, we offer to solve security and privacy concerns.

## IV. Par Deployment in a Real Context

This section presents the strategies used to implement the proposed solution, describing the procedures used to collect and treat sensor data. Besides, it explains the process of deployment PAR in a real context of an Intensive Care Unit, presenting the results obtained by using the solution, which makes it possible to process data from different unobtrusive sensors in real-time.

### A. Deployment Context

**FIGURE 9.**
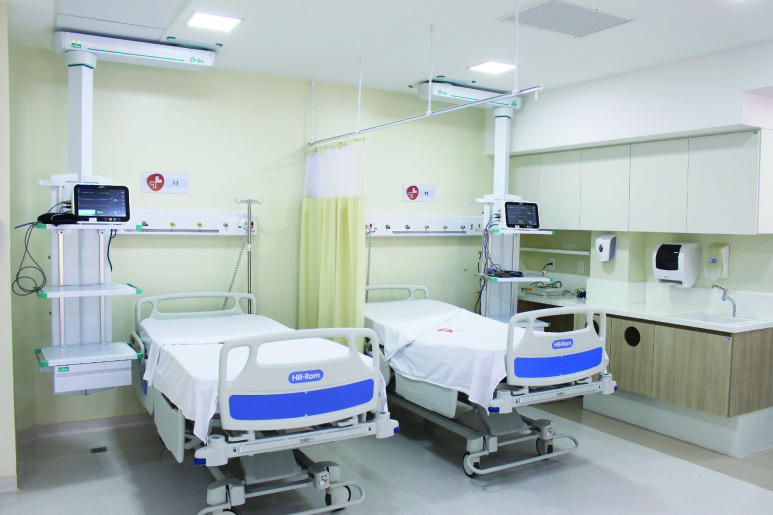
Part of the ICU beds being monitored with PAR [55].

The solution proposed in the previous section was implemented in the context of an Intensive Care Unit (ICU) in the city of Natal, in the state of Rio Grande do Norte, Brazil. The ICU has a capacity for 12 critical beds, each bed being equipped with a multiparametric monitor, which receives the data collected through sensors connected to the inpatient. Figure 9 shows two of the twelve installed beds, properly equipped with multiparametric monitors.

The ICU is also equipped with a Monitoring Center to which each monitor is connected via a Virtual Local Area Network (VLAN) [56] dedicated to the monitors and the Monitoring Center. The equipment installed in the ICU is from the same manufacturer, and the monitors are also of the same model. Each monitor communicates with the Central through the HL7 protocol and sends, every 5 minutes, personal data such as name, sex and date of birth, the patient’s health data being collected through the sensors.

Various patient health signs are monitored by sensors that are connected to the patient and monitors, including arterial blood pressure, respiratory rate, heart rate, non-invasive pressure (systolic, diastolic, and mean), oxygen saturation ($SpO_{2}$), ST segments of the ECG, levels of $CO_{2}$ (capnography [57]) and, finally, the values of room temperature and the patient’s body. This wide variety of data collected about the patient is vital for treating the patients that are being monitored.

### B. Deployment Process

Considering the context of the aforementioned ICU, the PAR implantation process took place through steps that involved the different aspects: configuration of the cloud environment prepared to manage IoT data; configuration of the network communication of the monitoring devices; configuration of the Gateway responsible for receiving data from devices and sending it to the server in the cloud; and the configuration of the components that will process the data and make it available for viewing. The details of the implementation, according to these aspects, are described below.

Initially, the configuration of the cloud infrastructure that is responsible for receiving, processing and making health data available was carried out. For this, the Message Queue Telemetry Transport (MQTT) protocol was used. MQTT uses a publish/subscribe model and is suitable for communication between restricted devices [58], such as health data monitoring devices.

Once collected, the sensor data are sent to the MQTT broker, which allows the connection between agents and data managers through automatic communication between them. That way, whenever a new agent publishes an association request for the topic of their data type, all subscribed managers receive the message and respond to the membership request [59].

After setting up the MQTT broker with the agents and managers who will act in the communication, the configuration step of the multiparametric monitors and the monitoring center began, aiming to enable the communication between the monitors and the sending of the data collected by the sensors to the monitoring center. For this, a secure and exclusive VLAN was created to include these devices in the hospital’s internal network.

Once the MQTT broker is configured and available to receive the data and the monitors are connected in a network, sending patient data to the monitoring center, it is necessary to configure a component responsible for the communication between the ICU devices and the MQTT broker. This component is the Gateway, installed on a Raspberry Pi 3 Model B + [60] and configured on the monitors’ VLAN. In this case, the monitoring center receives data from all monitors connected to the network and sends it to the Gateway. For this, it was necessary to inform the central the IP address of the Gateway and which port is listening to receive the data. In this case, port 2575 is used, the standard for communication using the HL7 protocol. In turn, the Gateway sends the data to the cloud server through a virtual network interface that has access to the internet. The Figure 11 shows the Raspberry Pi 3, which has the Gateway installed, connected to the exclusive ICU VLAN via the ethernet port.

**FIGURE 10.**
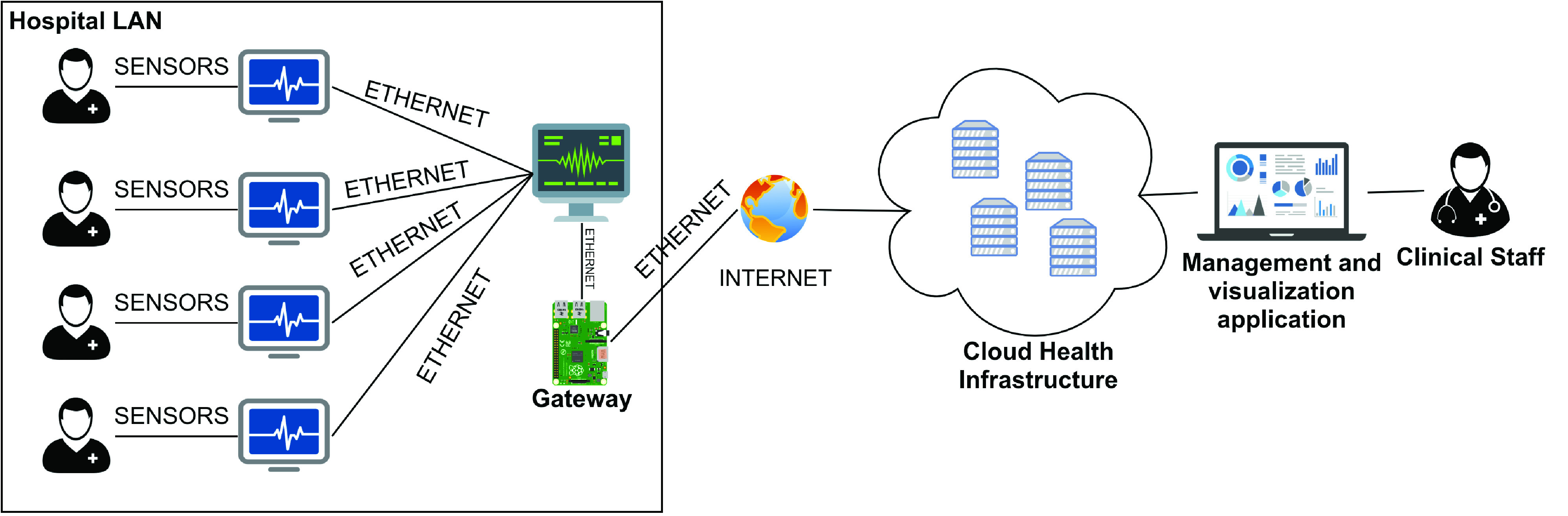
The connections between sensors, multi-parameter monitors and the solution for remote monitoring in the ICU.

**FIGURE 11.**
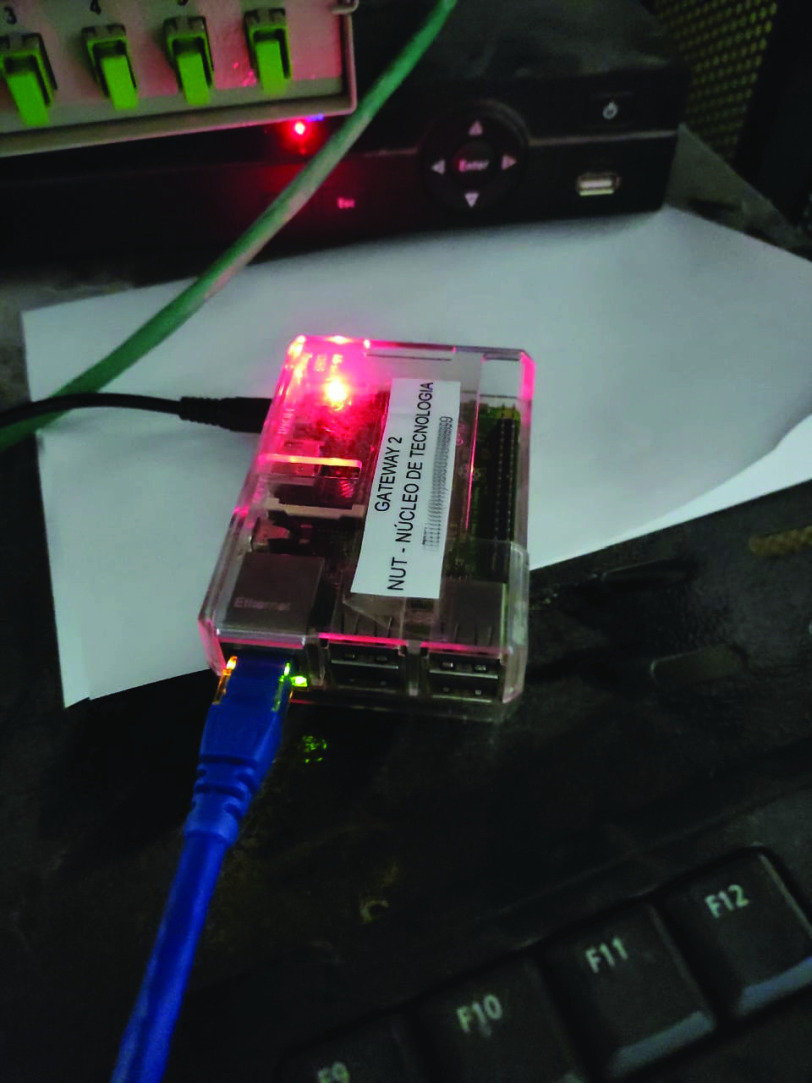
The Raspberry Pi 3 where the PAR Gateway was installed, connected to the ICU VLAN.

Thus, with the Gateway connection to the monitoring center and the MQTT broker properly established, this center’s data starts to be sent to the broker. From there, the sensor data undergo a treatment process until it becomes available. This process happens through the reading and writing of the topics of the MQTT broker, carried out by three main agents, described below:

• IoT data collector: This component is responsible for interpreting the HL7 packages, creating objects with the values received from the sensors connected to the patients and writing them in a new topic of the MQTT broker, contemplating the RAH Remote Patient Monitoring module, proposed in the previous section;
• Intelligence and emergency: This component includes RAH’s Emergency and Crisis Management, as it is included in the topic in which IoT data collector writes and uses Artificial Intelligence (AI) techniques to verify the signals’ values according to the safety limits established by clinical protocols. In case of verification of values outside the established limits, this component is also responsible for sending alerts to the clinical team;
• Data visualization and management: This component includes RAH’s Patient Health Data Management and Patient Health Management modules. Through the data visualization and management component, it is possible to manage the patients’ health data, especially the health situation of each one, and access the history of the sensors’ monitoring data, enabling the definition of safety limits according to the protocols used in the ICU.

Figure 10 illustrates the connections between the components of PAR implantation in the context of the ICU mentioned above. It is possible to identify how the monitors and the central communicate with each other and with the Gateway, as well as the communication of this with the MQTT broker, which is in the cloud until the data is made available to the ICU clinical team.

### C. Deployment Costs

The deployment of systems can present a very high cost, which often discourages the development of promising solutions. This problem usually happens in small and medium-sized institutions, which do not have much capital for large investments. However, this article presents an alternative proposal based on low-cost components, such as raspberry pi, and a relatively common and simplified cloud infrastructure capable of delivering the services needed to execute the proposed solution. A cost simulation in dollars of this infrastructure can be seen in Table 2.

**TABLE 2:** Cloud Services and Raspberry Pi 4 Costs

Cores	RAM	Storage	Price/Month	Raspberry	Total
4	8 GB	40 GB	~$191,26	~$35	~$226,26

### D. Deployment Results

PAR was implemented in December 2019 and is in full operation until the present day. As of this article’s writing of this article, approximately 139 patients were monitored remotely through the platform, which has data from 22 different types of health data collected from patients. Health data for each patient is sent by multi-parameter monitors and collected by the PAR Gateway every 5 minutes. The use of the solution brought gains in various aspects of the day-to-day of the ICU, among them:

• Remote monitoring and speed in managing conduct: the ICU physician on duty can monitor all hospitalized patients from anywhere, in addition to receiving alerts through notifications on their cell phones, facilitating the distance guidance of conducts that must be taken with each patient, according to their clinical status;
• The abolition of paper and manual recording of vital sign data: previously, nursing technicians periodically collected data from each patient, and this record was done by hand. Through PAR, the registration is done automatically and digitally, facilitating access to information and removing the need to move to the patient’s bed to record the data;
• The decrease in direct contact with the patient and the medical team’s exposure: the medical team started to decrease the frequency with which it goes to the patient’s bed since his health data can be checked at any time through the platform online;
• The ease of accessing data collected through the discrete sensors connected to the patient: any sensor that monitors the patient and is also connected to the same VLAN to which PAR is connected can have its data collected through the platform, facilitating access to the clinical data of the patient. Besides, if new sensors are used in this monitoring, the application will have access to the data automatically without changing the implanted solution’s structure.

In the context of the Corona Virus Disease (COVID-19) pandemic, the ICU where PAR was implanted is being used to treat patients with the disease. This disease’s nature makes it necessary to have minimal contact with infected patients since direct contact with infected patients represents risks for the clinical team [61] and, therefore, the remote monitoring solution has become a great ally in the fight against the disease. When using the platform, members of the ICU clinical team do not require constant direct contact with the patient to check vital signs, reducing the risk of contagion, especially among health professionals who are in the frontline of fighting the disease.

Health data collected, processed and analyzed through PAR can be made available in different formats. These data can be visualized through dynamic graphs with historical series of each vital sign monitored in each patient; complete reports for a specific period chosen by the user; and by exporting spreadsheets in $XLSX$ format.

**FIGURE 12.**
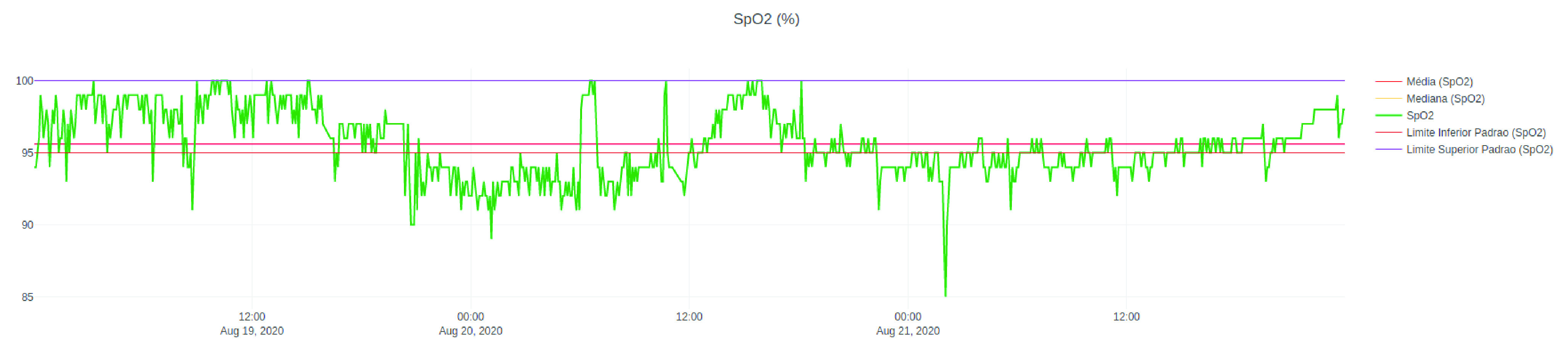
History from 19 to 21 August 2020 of $SpO_{2}$ of a patient admitted to the ICU where PAR is implanted.

**FIGURE 13.**
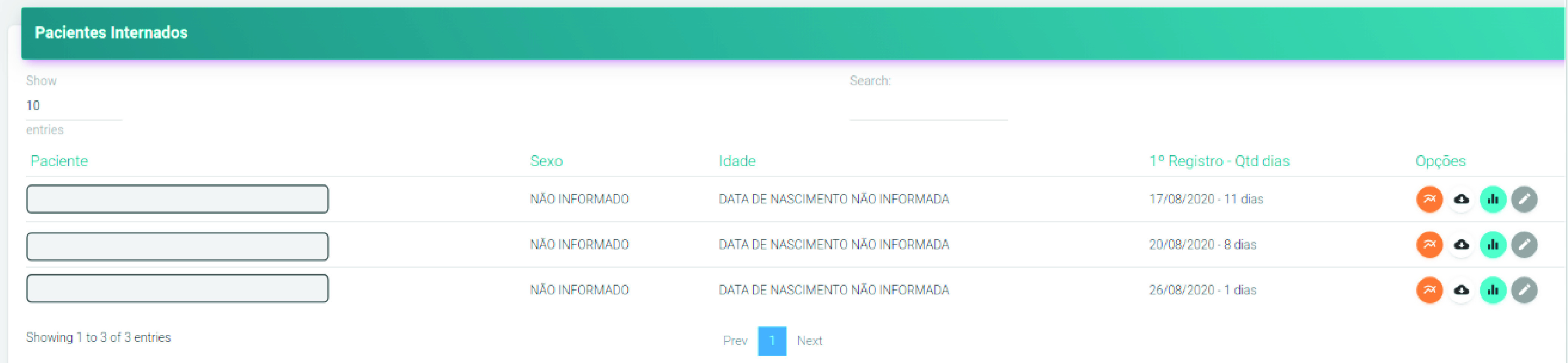
List of patients admitted to the ICU at that time.

**FIGURE 14.**
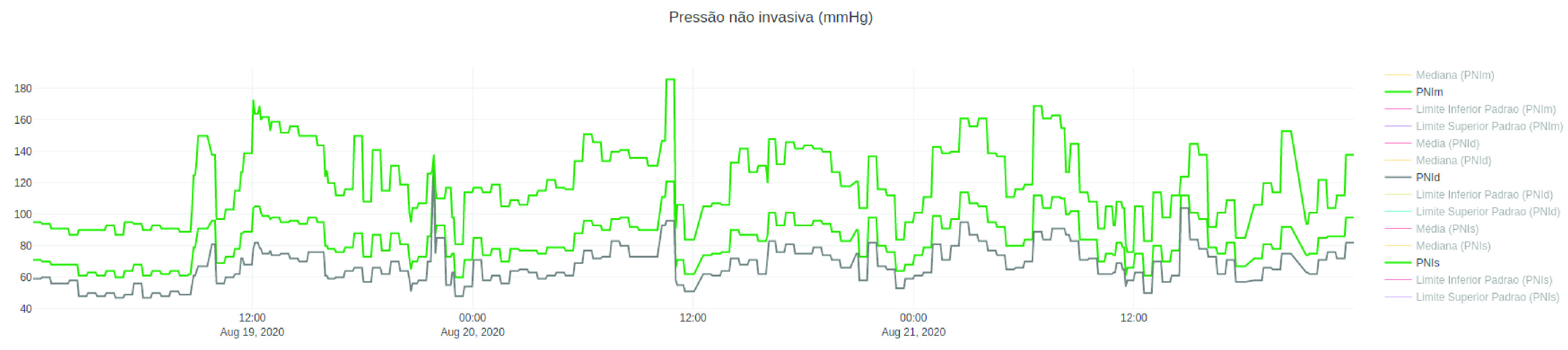
History from 19 to 21 August 2020 of $NBP$ of a patient admitted to the ICU where PAR is implanted.

Figure 13 shows the system’s home page, which consists of the list of patients admitted to the ICU at that time. The table has the columns of name, sex, and age of each hospitalized patient, as well as the number of days that each of them is hospitalized in the ICU. In this Figure, the names of the names were hidden to maintain their anonymity. From the list of patients, it is possible to navigate to the page with the data of the patients’ vital signs displayed in graphs. The system automatically generates a graph for each monitored signal in each patient, facilitating the clinical team’s visualization. Figures 12 and 14 show examples of graphics generated by the application.

Figure 12 shows a line graph containing the $SpO_{2}$ history, between 19 and 21 August 2020, of a patient admitted to the ICU where PAR was implanted. The graph includes five lines. The green line consists of the values of $SpO_{2}$ obtained over time, while the red and yellow lines represent, respectively, the mean and median of the values of $SpO_{2}$ in that period of time. Finally, the purple and pink lines represent the maximum and minimum safety limits, respectively. It is important to note that safety limits are defined by the ICU clinical team and can be defined in a standard way for a group of inpatients or customized according to the clinical status of each patient.

Figure 14 shows a line graph containing the systolic, mean, and diastolic Non-invasive Blood Pressure ($NBP$) between 19 and 21 August 2020 of a patient admitted to the same ICU. The graph also shows lines with the mean, median values, and the maximum and minimum safety limits for the $NBP$.

**FIGURE 15.**
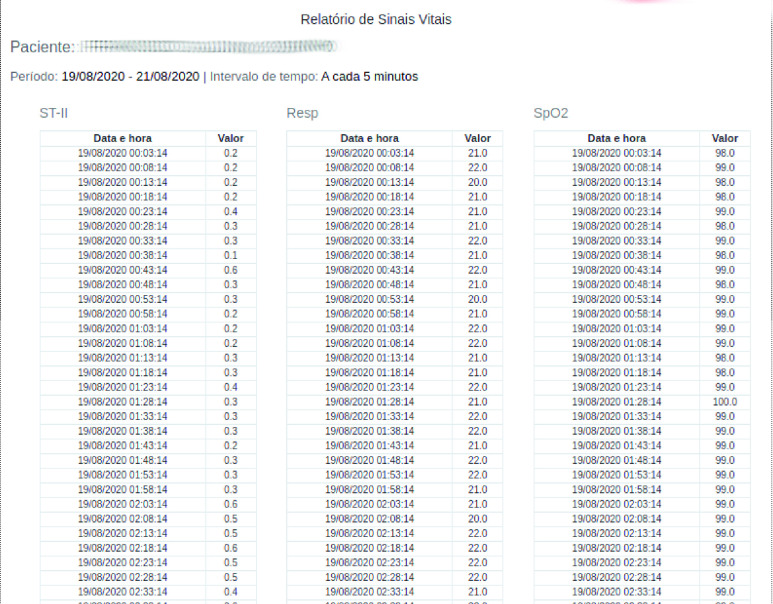
Historical report between August 19 and 21, 2020 of the $ST-II$ segment, breathing ($Resp$) and $SpO_{2}$ of a patient admitted to the ICU where PAR was implanted.

Figure 15 shows part of a vital signs report of a patient admitted to the ICU and monitored with PAR, generated with the history between the 19th and 21st of August 2020 of the $ST-II$ segment, breathing ($Resp$) and $SpO_{2}$.

The platform has been in full operation on an uninterrupted basis since it was fully deployed, still in 2019. Several patients have already been monitored by PAR, and data from different sensors were collected without the need for any changes to the platform. The implanted solution is capable of automatically obtaining the data collected by the different sensors that monitor the patients, enabling better working conditions for the clinical team, lower costs for the hospital and better assistance for people who are hospitalized.

Finally, it is important to highlight that PAR is a solution that is being useful in the context of the COVID-19 pandemic, but it can also be applied and of great value in different contexts, such as in the process of dehospitalization. Promoting remote monitoring, PAR is a solution that can support this process, allowing patients who are in home-care environments to be monitored by the clinical team remotely and in real time.

## V. Conclusion and Future Works

This paper extended previous work related to an IoT-based healthcare platform for remote monitoring of patients in critical condition, showing how this solution is adequately flexible to be used in contexts involving pervasive continuous health monitoring with the support of unobtrusive devices. Moreover, we reported the experience involving the development and deployment of a remote monitoring solution in an ICU of a Brazilian hospital, which was based on our approach.

This experience offers an important foundation for expanding our approach for other contexts involving critical patient monitoring. We hope that these results will attract interest in the expansion of researches on this topic. As a result, we will evolve towards filling in the current gaps and limitations hindering the adoption of continuous health monitoring supported by unobtrusive sensors.

Finally, as future works, we expect to integrate machine learning algorithms to predict risky events and take action rapidly in order to maximize treatment effectiveness. Our approach is currently cloud-oriented, so we believe that a relevant evolution could borrow some fog computing architectures to improve its proactive ability and provide fast alerts.
